# Predicting response to early reading intervention for children at risk for dyslexia – the importance of pronunciation accuracy

**DOI:** 10.1007/s11881-026-00388-9

**Published:** 2026-08-11

**Authors:** Anna Steenberg Gellert, Dorthe Klint Petersen, Carsten Elbro

**Affiliations:** Centre for Reading Research, University of Copenhagen, Copenhagen, Denmark

**Keywords:** Prediction, Intervention response, Dyslexia, Second language, Pronunciation accuracy

## Abstract

This study aimed to examine the extent to which pretest measures predicted response to an early reading intervention for children at risk for dyslexia. Predictors were studied in both first-language (L1) speakers and second-language (L2) learners. The pretest measures assessed various reading-related abilities, including pronunciation accuracy. Data from the experimental group in a trial of a reading intervention of 76 sessions for first-grade students at risk for dyslexia were analyzed. Outcomes were examined for 101 participants, including 68 L1 speakers and 33 L2 learners of Danish, based on their posttest performance on reading of words and non-words. Pretest measures of decoding, letter knowledge, letter-sound knowledge, phonological awareness, rapid automatized naming, vocabulary, and pronunciation accuracy were evaluated as predictors of reading outcomes. The strongest predictors of children’s reading outcomes were phonological awareness, letter knowledge, letter-sound knowledge, and pronunciation accuracy. A relatively good classification of children with subsequent poor or adequate response to the intervention could be obtained based on pretest measures of letter-sound knowledge and pronunciation accuracy. Pronunciation accuracy added uniquely to the prediction of overall variance in reading outcomes for L1 speakers and L2 learners and to the correct early classification of children as poor or adequate responders. The results suggest that an acceptable level of prediction of at-risk children’s response to early reading intervention can be obtained by means of a few pre-intervention measures. This study provides evidence for the added predictive value of a measure of pronunciation accuracy for L1 speakers and L2 learners.

## Introduction

Some children experience significant difficulties learning to read in the early school years. These children often have weak pre-reading skills and are at increased risk of developing persistent reading difficulties. Thus, low levels of early decoding abilities, letter knowledge, phonological awareness, and rapid automatized naming (RAN) have been shown to be predictive of later reading difficulties (e.g., Gellert & Elbro, 2018; Poulsen et al., 2017; Thompson et al., 2015). Furthermore, children with speech-sound difficulties that persist in school-age have been found to be at particular high risk (e.g., Burgoyne et al., 2019; Tambyraja et al., 2020).

Fortunately, early intervention can prevent or reduce later reading difficulties for many at-risk children. Early interventions with documented effects for such children are intensive and take place over an extended period in small groups or individually (Wanzek et al., 2016). These interventions typically include systematic and explicit instruction in letter-sound correspondences, letter-sound analysis and blending for decoding words (e.g., Hatcher et al., 2006; Solheim et al., 2018; see Hall et al., 2023). However, at-risk children do not respond similarly to a given program. Thus, even for early reading interventions that are documented to be generally effective, a significant proportion of the participants do not respond well to the instruction (e.g., Greulich et al., 2014; Hatcher et al., 2006).

Research into the prediction of at-risk children’s response to early reading intervention is important, as this may improve our knowledge about how individual differences account for differential intervention outcomes and help to understand why some children are more resistant to interventions than others (Tilanus et al., 2019). From a practical perspective, knowledge of factors related to intervention responsiveness can aid in deciding which children are in greatest need of maximal support and intensive intervention (Lam & McMaster, 2014).

Previous research into the prediction of response to early reading intervention has indicated that children with low levels of phonological awareness not only have an increased risk of becoming poor readers but also tend to be less responsive to early reading intervention (Lam & McMaster, 2014). Children with related phonological difficulties such as speech-sound difficulties have been found to be particularly at risk for later reading difficulties (Walquist-Sørli et al., 2025; see further below). However, to our knowledge pronunciation accuracy has not been investigated as a possible predictor of at-risk children’s response to early reading intervention. This was done in the present study.

Furthermore, prediction of responsiveness to early reading intervention has primarily been investigated among children with the language of instruction as their first language (L1) (e.g., Greulich et al., 2014; Hatcher et al., 2006; van Uittert et al., 2022). More research into prediction of response to early reading intervention for L2 learners is important as these children presently constitute a relatively large group in many school settings. Therefore, the present study investigated how response to early reading intervention can be predicted for L1 speakers as well as L2 learners at increased risk for dyslexia.

## Predicting response to early reading intervention

Researchers have used different ways to conceptualize and quantify response to intervention. Following Grigorenko (2009), these ways can broadly be grouped into methods using the post-intervention outcome status, the rate of growth (or change while responding to intervention), or a mixture of both. The outcome-based methods (which are used in the present study) are based on specific thresholds such as reaching a particular score on a standardized achievement test or a criterion-referenced benchmark. Children scoring below such a threshold are often referred to as poor responders (e.g., O’Connor et al., 2010; Lam & McMaster, 2014).

A factor can be classified as predictive of responsiveness if pretest performance levels accurately differentiate between students who respond or do not respond to intervention (Lam & McMaster, 2014). Previous research into the prediction of at-risk children’s response to early reading intervention has identified a few rather consistent predictors of responsiveness. Particularly, phonological awareness has been found to be a critical indicator of intervention response in this population (see reviews by Al Otaiba & Fuchs, 2002; Lam & McMaster, 2014; Nelson et al., 2003). The most recent review of predictors of responsiveness to early reading intervention also highlighted skills in word identification, alphabetic principle (i.e., letter-sound correspondence, word attack, and decoding), and fluency as relatively consistent predictors across the studies while factors identified as generally not predictive included vocabulary and language (Lam & McMaster, 2014). In early reading interventions for non-readers, measures of reading skills are not likely to be useful as predictors since all the participants will be at floor level. Nevertheless, as it is well established that young children’s performance on measures of phonological awareness and letter knowledge predicts early reading development in alphabetic orthographies (e.g., Landerl et al., 2021), it is not surprising that these factors also have emerged among the predictors of response to early reading intervention.

However, although children with related phonological difficulties such as speech-sound disorders have been found to be particularly at risk for later reading disorders (cf. Walquist-Sørli et al., 2025), pronunciation accuracy has not been included as a possible separate predictor of response to early reading intervention in the reviews mentioned above and apparently neither in more recently published separate studies. Indeed, to our knowledge, pronunciation accuracy has so far only been investigated and documented as a predictor of at risk-children’s response to training in *phoneme awareness* (Elbro & Petersen, 2004; see next section). Therefore, it is unknown whether a pretest measure of pronunciation accuracy would also be predictive of response to *reading* intervention.

Furthermore, research in prediction of L2 learners’ response to early reading intervention is scarce and the results not very promising. At least two groups of researchers have attempted to identify predictors of responsiveness for L2 learners and have failed to find any (Linan-Thompson et al., 2006; O’Connor et al., 2010). Linan-Thompson et al. (2006) investigated the prediction of outcomes of a reading intervention conducted in either English or Spanish for first-grade English language learners whose home language was Spanish and who were at risk for reading difficulties. Before intervention Linan-Thompson and colleagues examined the children’s language proficiency and performance on tests of word attack and passage comprehension in English and Spanish. Based on the pretest measures, Linan-Thompson et al. were not able to identify any criteria that would indicate which participants would later be categorized as responders or non-responders.

O’Connor et al. (2010) investigated the effects of an early reading intervention for a broader group of kindergartners with low reading and language levels, about half of whom were English language learners. Before intervention O’Connor et al. examined the children’s rapid letter naming, initial sound fluency, vocabulary, English language development, and IQ. They found that none of these factors were predictive of the children’s response to intervention. This result is partly in line with Lam & McMaster who concluded that measures of vocabulary and language generally were not consistent predictors of response to early reading intervention (see above). However, the failure to identify predictors of response to early reading intervention in children with limited knowledge of the language of instruction could also reflect broader challenges of predicting reading outcomes in such children.

Indeed, in a recent review of studies of assessments conducted among L2 learners to predict reading achievement in their L2, Lai et al. (2026) reported relatively week evidence of criterion validity of constructs like letter knowledge and RAN. On the other hand, significant evidence of criterion validity was reported for measures of decoding and phonological awareness. However, it is unclear whether any reading-related measures can predict L2 learners’ response to early reading intervention.

## The predictive value of pronunciation accuracy

Children with speech-sound difficulties that persist into school-age and the onset of reading instruction are at increased risk for later reading difficulties (Burgoyne et al., 2019; Tambyraja et al., 2020).

Another at-risk group is formed by children with a family risk of dyslexia. Children from this group who go on to develop reading difficulties show weaknesses in a range of phonological areas, not only with phonological awareness, but also with pronunciation accuracy (e.g., Carroll et al., 2014; Elbro et al., 1998; Snowling & Melby-Lervåg, 2016).

It is very likely that the influence of pronunciation accuracy on early reading development is mediated by phonological awareness (Anthony et al., 2011; Elbro & Pallesen, 2002). Burgoyne et al. (2019) found that children with speech difficulties scored significantly lower than their peers at school entry on measures of letter-sound knowledge, phoneme awareness, and word reading. These differences remained significant after six months in school. A mediation model demonstrated that the relationship between speech difficulties and later reading was entirely mediated by phoneme awareness. Tambyraja et al. (2020) even reported a unique contribution from pronunciation accuracy on initial reading development after accounting for children’s age, general language abilities, socioeconomic status, *and* phonological awareness. However, this study followed children in kindergarten, Grade 1 and Grade 2, all of whom were diagnosed with speech-sound disorder.

Further evidence for at least an indirect relationship between pronunciation accuracy and response to early phonological awareness intervention comes from intervention studies. Elbro and Petersen (2004) reported on the long-term effects of a phoneme awareness intervention for Danish kindergarten children at family risk for dyslexia. The authors found that individual gains in phoneme awareness in the experimental group were predicted by the children’s initial pronunciation accuracy.

In sum, pronunciation accuracy has been found to predict reading difficulties in at-risk children as well as response to phoneme awareness training in at-risk children. It seems reasonable to hypothesize that individual differences in pronunciation accuracy may also predict individual outcomes of an early reading intervention for at-risk children. However, to our knowledge, this has not been investigated previously. The present study aimed to test this hypothesis.

## Research questions

This study aimed to investigate how well pretest measures of reading-related abilities predict at-risk children’s responses to an early reading intervention. It allowed for a comparison of predictors in L1 speakers and L2 learners. Our research questions were:

(a) To what extent can overall variance in reading outcomes following an early intervention be predicted by pretest performance on measures of decoding, letter knowledge, letter-sound knowledge, phonological awareness, RAN, and vocabulary among at-risk children receiving instruction in their L1 or L2?
  (b) Does the inclusion of a measure of pronunciation accuracy account for additional variance beyond the other predictors among L1 speakers and L2 learners, respectively?
(a) To what extent can pretest performance on the most predictive measures classify children with subsequent poor or adequate response to the intervention?
  (b) Does the inclusion of a measure of pronunciation accuracy add to the early classification beyond the other predictors?

## Method

### Design

The present investigation is an analysis of data from a recent study examining the effects of a reading intervention for first-grade students at risk for dyslexia, using an experimental design with an intervention group and a control group (Gellert et al., 2024). This analysis uses pre- and post-intervention data from the experimental group only, including previously unpublished pre-intervention data collected specifically for the current investigation.

Tests of reading of words and non-words were administered to the children at posttest and served as the primary outcome measures for the intervention. At pretest we administered tests of word reading, dynamic decoding, letter knowledge, letter-sound knowledge, phonological awareness, RAN, vocabulary, and (particularly for the present investigation) pronunciation accuracy.

In the following sections, we provide information about the participants, the intervention, and the measures of interest for the current analysis. The materials and measures were in Danish, a language that shares many similarities with English. Both Danish and English are Germanic languages with complex syllable structures and deep orthographies (Elbro, 2005; Seymour et al., 2003). Previous Danish studies investigating predictors of reading development and difficulties have yielded findings (e.g., Elbro et al., 1998; Juul et al., 2014; Poulsen et al., 2017) that closely parallel those reported in English-language research.

### Participants

Children at increased risk of dyslexia from a total of 34 schools from 12 different municipalities participated. To identify at-risk children, we conducted testing in all kindergarten classes at the participating schools. A total of 252 children were selected for the study among the lowest performing at their schools based on their total score of an assessment of increased risk for dyslexia described in more detail below.

Of these 252 children, 136 attended schools that had randomly been assigned to the experimental condition while the remaining 116 children attended schools assigned to the control condition. After attrition, pretest and posttest data were available from 111 participants in the experimental condition and 96 in the control condition.

In the current investigation, only data from 101 of the 111 participants in the experimental condition were included, as data from the pronunciation accuracy measure of special interest for this investigation were not available for the remaining ten children. This reduction was partly because five of these children were not available for the last part of the testing. In one case, the quality of the recording was too low to be used for assessment of pronunciation accuracy. This assessment was also not possible for another four children because they did not name any of the pictures meant to elicit their pronunciation (see description of the task below).

Sixty-eight of the 101 participants spoke Danish as their L1, while 33 spoke Danish as an L2 according to information provided by the schools. The average age of the participants was 6 years 11 months at pretesting in the end of kindergarten (which is the usual age at this point in Danish schools).

### Reading intervention

The intervention program was developed specifically for first-grade students at risk for dyslexia in a recent study (Gellert et al., 2024). The program comprised 76 sessions of instruction in basic letter-sound knowledge, letter-sound analysis, blending for reading short, regular words, and scaffolded reading of short texts. All the sessions were scripted in manuals with detailed instructions and modelling of the procedures and strategies. The teaching was conducted by the local reading teachers at the schools and carried out over 19 weeks with 4 weekly sessions in small groups of max. 5 children.

After completing a brief introductory course provided by the project team, the reading teachers implemented the first phase of the intervention program during the fall term of Grade 1. This phase consisted of nine weeks of phonics instruction, delivered in four sessions per week. During this period, children worked with letter sounds to develop functional letter-sound knowledge and with letter-sound analysis and blending for decoding and spelling short words. Instruction incorporated embedded picture mnemonics (Shmidman & Ehri, 2010) and connected phonation techniques (Gonzalez-Frey & Ehri, 2021). Following a second introductory course, the teachers delivered the second phase of the program in the spring term. This phase involved ten weeks of training focused on teaching children a phonics-based strategy for reading short texts composed of phonetically decodable words as well as spelling.

The children in the control condition received the instruction they would normally get at their respective schools, including any special interventions in small groups (business as usual).

In the recent trial of the intervention program, medium to large effects of the intervention were found on children’s reading accuracy with untrained words (*d* = 0.47) and non-words (*d* = 0.54) (see description of these primary outcome measures below). Further details about the program and the effects are provided by Gellert et al. (2024).

### Measures

The predictor measures were administered at the end of kindergarten and the primary outcome measures after the intervention at the end of Grade 1. The measures described below were administered individually in quiet rooms within the schools and recorded by a trained research assistant or by trained students of speech-language pathology or linguistics. (Reliability estimates for study measures and corresponding source references are summarized in Table 6 in the Appendix).

#### Predictor measures

As mentioned, an assessment of increased risk for dyslexia (*Risk Test for Dyslexia*) developed by Gellert and Elbro (2018) was used to identify children at increased risk of dyslexia. This assessment included the first three pretests in this study. Children who performed poorly on the Risk Test for Dyslexia completed the additional tests on another day.

### Word reading

This test of word reading is the first subtest in the Dyslexia Risk Test at the end of kindergarten where it is used as a screener to determine whether children can read at all. If the child cannot read any words correctly, the test administrator proceeds to the next subtests in the Dyslexia Risk Test. If a child can read one or more words correctly, he or she is generally not considered at risk for dyslexia, and the test administrator only proceeds to the next tests in cases of doubt.

Word reading is assessed by means of 30 single, real words to be read aloud (Elbro & Petersen, 2004). The words are all orthographically regular and between two and nine letters long. The score taken is the number of words read correctly.

### Letter knowledge

The second subtest in the Risk Test for Dyslexia is a traditional test of letter name knowledge developed by Borstrøm and Petersen (2006). In this test the child is presented with all the 29 letters (in Danish) in a non-alphabetic order, first in the upper case and then in the lower case and asked to name them. The internal consistency (Cronbach’s alpha) was 0.91 and 0.92 for upper- and lower-case letters, respectively (Gellert et al., 2024). The scores for upper and lower case were highly correlated (0.93). They were standardized and averaged into a composite measure for analyses reported in the Results section.

### Dynamic decoding

The third subtest in the Risk Test for Dyslexia is a dynamic test of decoding developed by Elbro et al. (2012). This test employs artificial letters and non-verbal gestures for instruction as it was constructed to reduce the influence of vocabulary and amount of schooling. This test has three parts:

1. *Learning novel letters.* The child is presented with three novel letter shapes and their sounds: ╔ = /s/, ◊ = /m/, and ◘ = /α/ as in *calm*. The three letter shapes are used in a paired associate learning task with corrective feedback. The score taken is the number of correct letter sounds (max 30).
2. *Learning to read by blending*. The child is shown how the three new letters are used to form simple CV and VC non-words, e.g. ╔ ◘ = [sα]. The experimenter demonstrates sound synthesis by gradually moving single letter cards together. The child is asked to make similar blends of four other two-letter non-words. Corrective feedback is provided for a maximum of five trials with the four non-words. If a criterion of all four non-words named correctly in two consecutive trials is not met, testing is discontinued. The score is the number of the non-words named correctly (max 20).
3. *Independent reading*. If the criterion is met in part 2, the child is presented with 12 new words made up of combinations of the three letters, e.g., ◊ ◘╔ ◘ (*masa*) and╔ ◊ ◘ ◊╔ (*smams*). The score is the number of correctly read non-words (max 12).

Cronbach’s alpha for the three parts of the dynamic test of decoding was 0.81, 0.96, and 0.93, respectively, in a previous study with kindergarten children (Gellert & Elbro, 2018).

A dyslexia risk score is calculated by adding the composite score from the measure of letter knowledge to the combined score from parts 2 and 3 of the dynamic assessment of decoding. A total score of 25 or below indicates an increased risk of dyslexia (Gellert & Elbro, 2016).

### Letter-sound knowledge

Letter-sound knowledge was assessed using a test developed for a previous study by Elbro and Petersen (2004). In the first part of this test, the child was presented with 12 consonant letters. Following a practice item, the child was asked to produce the sound associated with each of the remaining 11 letters. In the second part, the examiner articulated each letter sound, and the child was instructed to point to the corresponding letter. Scores were based on the number of correctly produced sounds (part 1) and correctly identified letters (part 2). Cronbach’s alpha coefficients were 0.88 and 0.84 for parts 1 and 2.

### Phonological awareness

Two subtests developed for kindergarten children in a previous longitudinal study (Gellert & Elbro, 2017a) were used to assess phonological awareness. The first subtest assessed syllable identification and included five test items in which the child was presented with six illustrations of common words (e.g., *giraffe*). The child was first asked to name the pictures and, if necessary, was corrected by the test administrator to ensure the child knew the words. Then, the child was asked to point to the picture of the word that began with a specific syllable (e.g., *gi-*).

The second subtest assessed initial sound identification. The child was again presented with six illustrations of common words (e.g., *boy*), which the test administrator named. The test administrator then pronounced a sound, e.g., /b/, and asked the child to point to the picture of the word that began with that sound. The child was presented with a maximum of 15 tasks, arranged in increasing difficulty based on results from Gellert & Elbro (2017a). The score was the number of correctly answered items. If the child answered incorrectly on three consecutive tasks during the second subtest, the test was discontinued. Cronbach’s alpha for the full test was 0.86.

### RAN

This test was adapted from a previous study with kindergarten children (Juul et al., 2014). The child was asked to name five rows of five stylized objects (car, scissors, heart, ice, sun) as quickly as possible, 35 objects in total. The same five objects were repeated in random order. The test score was calculated as the number of correctly named objects per second. The correlation between the original version of this test and a test of RAN with digits was reported at *r* = .63 which is a lower bound estimate of the reliability (Juul et al., 2014).

### Vocabulary

To obtain a measure of the children’s vocabulary breadth in Danish, we used an excerpt from an extensive picture-naming test developed for a previous longitudinal study (Gellert & Elbro, 2018). In this test the child was asked to name color photographs using a single word, e.g., *strawberry* and *octopus.* Based on kindergarten results from the previous study, we chose 35 out of the original 84 test items to adequately represent a suitable range for vocabulary assessment in the current sample. Among the 35 items we selected 16 items featuring target words that were relatively phonologically complex and likely to elicit pronunciation errors for children with speech-sound difficulties. (A list of these 16 words is provided in Table 7 in the Appendix). After having been asked to name the pictures of the first 19 items, the child was presented with these 16 items and provided with a prompt if he or she could not name the picture (see below). The score for vocabulary was the number of correctly named pictures without prompting (max. 35). Cronbach’s alpha for the test excerpt used was 0.92.

### Pronunciation accuracy

A pronunciation accuracy measure was developed and used with the final 16 vocabulary items (see above). For each item, the child first named the picture, as with the previous 19 items. If the child could not correctly name a picture independently, a prompt was given to elicit a pronunciation of the target word. The prompt was provided by means of the character Jeppe the Helper, who was illustrated flying in on a bird and, through the test administrator, said part of the word the child was expected to produce, e.g., [kæ] for *kangaroo.*

Credit was given if the target word was pronounced acceptably, i.e., in accordance with standard pronunciations listed in the comprehensive Danish Dictionary of Pronunciation (Brink et al., 1991) and/or typical pronunciations for children of similar age. Two scores were derived from the pronunciation accuracy measure: (1) the percentage of acceptably pronounced words out of words correctly named without prompting, and (2) the percentage of acceptably pronounced words out of *all* words correctly named, both with and without prompting.

All pronunciations of the 16 words were transcribed by a trained linguist. The first author scored the pronunciations according to the criteria mentioned above. Uncertainties about the scoring were discussed and resolved between the first and the second author (who were both trained as speech-language pathologists).

#### Reading outcome measures

By the end of Grade 1, the children in the current sample were still in the early stage of reading development and had not yet achieved sufficient reading accuracy for significant progress in reading speed (Juul et al., 2014). Therefore, the outcome measures assessed reading *accuracy* rather than fluency.

### Word reading

The children received the same test of word reading as in kindergarten. The score is the number of words read correctly. In the previous longitudinal study by Gellert and Elbro (2018), the correlation between test scores from November of Grade 1 and May of the same school year was reported at 0.82. This provides a lower bound estimate of the reliability of the test.

### Non-word reading

This test with pronounceable non-words is parallel to the test of word reading (Elbro & Petersen, 2004). The non-words are matched individually to the real words in terms of length (number of letters) and syllable structure. The score is the number of non-words correctly read. Consistent with previous findings from first graders reported by Gellert and Elbro (2018), the scores of non-word reading correlated strongly with word reading (0.86) in the present sample. The scores from the lists of words and non-words were standardized and averaged into a composite measure for some of the analyses reported below.

## Results

### Distributions and correlations

Table 1 provides descriptive statistics for the predictor measures administered in kindergarten and the outcome measures taken in Grade 1 separately for children with Danish as their L1 or L2.

**Table 1:** Means and standard deviations with pretest and posttest measures for participants with Danish as L1 and L2

Time	Measure	Max score	Mean (standard deviation)	
			L1 (*n* = 68)	L2 (*n* = 33)
Kindergarten	Word reading	30	0.00 (0.00)	0.03 (0.17)
	Letter knowledge 1: Upper case	29	17.01 (6.86)	15.45 (7.85)
	Letter knowledge 2: Lower case	29	14.51 (6.92)	12.85 (7.54)
	Letter knowledge (composite)		0.07 (0.94)	− 0.15 (1.06)
	Dynamic decoding 1: Learning novel letters	30	22.72 (6.06)	23.36 (5.47)
	Dynamic decoding 2: Learning to read	20	3.54 (3.18)	3.45 (3.85)
	Letter-sound knowledge 1: Producing letter-sounds	11	3.71 (3.33)	3.64 (3.49)
	Letter-sound knowledge 2: Identifying letters	11	6.90 (3.03)	5.88 (3.42)
	Phonological awareness 1: Syllable identification	5	4.04 (1.03)	3.94 (0.97)
	Phonological awareness 2: Phoneme identification	15	8.18 (3.69)	7.76 (3.49)
	RAN		0.78 (0.17)	0.77 (0.21)
	Vocabulary	35	25.62 (5.26)	14.91 (8.18)**
	Pronunciation accuracy 1: Acceptably pronounced words *without* prompting (%)	100	75.60 (15.40)	78.53 (23.99)
	Pronunciation accuracy 2: Acceptably pronounced words in total (%)	100	72.35 (15.16)	73.43 (22.39)
Grade 1	Word reading	30	8.71 (5.53)	11.27 (8.12)
	Non-word reading	30	9.16 (5.93)	12.30 (7.78)*
	Reading (composite)		− 0.14 (0.83)	0.29 (1.18)*

*p < .05; **p < .01

An expected strong floor effect was found on early word reading in kindergarten. To a lesser extent, the second part of the dynamic test of decoding (learning to read) also showed evidence of a floor effect. None of the participants were able to correctly read all the non-words in two consecutive rounds in this part of the dynamic test, and therefore they did not proceed to the third subtest. Accordingly, the results for this subtest are not included in the tables.

Further statistics for the measures of pronunciation accuracy are provided in Table 8 in the Appendix.

Independent-samples *t* tests indicated that the L1 group scored significantly higher than the L2 group on the pretest measure of vocabulary, *t*(99) = 7.95, *p* < .001. However, the L2 group performed significantly better than the L1 group on the posttest measure of non-word reading, *t*(99) = -2.05, *p* < .05, and the composite reading measure, *t*(99) = -2.12, *p* < .05. No significant differences were found between the two groups for the remaining measures.

Table 2 presents the intercorrelations among the pretest and posttest measures, shown separately for L1 speakers (below the diagonal) and L2 learners of Danish (above the diagonal). The pretest word reading measure was excluded from the correlation analyses and the regression analyses due to a lack of variability. To simplify these analyses, only the composite score for pretest letter knowledge was included.

For both language groups, the two pronunciation accuracy measures were strongly correlated (*r* ≥ .76).

**Table 2:** Correlations between measures in kindergarten and Grade 1 for L1 speakers (*N* = 66) in the lower triangle and for L2 learners (*N* = 33) in the upper triangle

		1	2	3	4	5	6	7	8	9	10	11	12	13	14
K	1. Letter knowledge (composite)	--	− 0.09	− 0.11	0.71**	0.73**	0.28	0.53**	0.28	0.11	0.49**	0.50**	0.60**	0.50**	0.56**
	2. Dynamic decoding 1: Learning letters	0.09	--	0.13	− 0.16	− 0.11	− 0.15	− 0.18	0.27	0.53**	− 0.14	− 0.25	− 0.06	− 0.01	− 0.04
	3. Dynamic decoding 2: Learning to read	0.02	0.09	--	0.02	0.02	0.07	0.16	0.10	− 0.12	0.25	− 0.10	0.04	0.17	0.10
	4. Letter-sound knowledge 1: Producing letter-sounds	0.71**	0.13	0.11	--	0.80**	0.30	0.46**	0.17	− 0.10	0.45**	0.40*	0.48**	0.41*	0.46**
	5. Letter-sound knowledge 2: Identifying letters	0.83**	0.16	0.10	0.69**	--	0.22	0.62**	0.33	0.20	0.51**	0.44*	0.56**	0.51**	0.55**
	6. Phonological awareness 1: Syllable identification	0.25*	0.24	− 0.19	0.47**	0.27*	--	0.24	0.04	− 0.13	0.47**	0.37*	0.41*	0.41*	0.42*
	7. Phonological awareness 2: Phoneme identification	0.46**	0.27*	0.00	0.59**	0.47**	0.50**	--	0.21	0.17	0.59**	0.49**	0.42*	0.33*	0.38*
	8. RAN	0.10	0.24	− 0.08	0.08	0.06	0.20	0.05	--	0.37*	0.40*	0.27	0.24	0.30	0.28
	9. Vocabulary	0.23	− 0.05	0.01	0.18	0.23	0.21	0.28*	0.12	--	− 0.08	− 0.14	0.18	0.20	0.20
	10. Pronunciation accuracy 1	0.15	0.21	− 0.02	0.16	0.34**	0.10	0.09	0.01	0.05	--	0.76**	0.55**	0.50**	0.53**
	11. Pronunciation accuracy 2	0.27*	0.19	0.02	0.19	0.41**	0.16	0.21	0.08	0.20	0.90**	--	0.50**	0.37*	0.45**
G1	12. Word reading	0.22	0.14	0.14	0.28*	0.29*	0.14	0.32**	0.04	0.18	0.20	0.22	--	0.92**	0.98**
	13. Non-word reading	0.14	0.18	0.13	0.34**	0.27*	0.26*	0.41**	0.01	0.12	0.27*	0.29*	0.86**	--	0.98**
	14. Reading (composite)	0.19	0.17	0.14	0.32**	0.29*	0.21	0.38**	0.03	0.15	0.25*	0.27*	0.96**	0.97**	--

*K* kindergarten, *G1* Grade 1, *PA* phonological awareness

* *p* < .05; ** *p* < .01

### Prediction of overall variance in reading outcomes for L1 speakers and L2 learners

Hierarchical linear regression analyses were conducted to address the first set of research questions concerning the extent to which pretest measures predicted overall variance in reading outcomes for L1 speakers (Table 3) and L2 learners (Table 4). In each analysis, the dependent variable was either posttest word reading or non-word reading. The first block of independent variables included pretest measures of dynamic decoding, letter knowledge, letter-sound knowledge (producing letter sounds and identifying letters), phonological awareness (syllable and phoneme identification), RAN, and vocabulary. To examine whether pronunciation accuracy could explain additional variance beyond the other predictors, the percentage of acceptably pronounced words without prompting (pronunciation accuracy 1) or in total (pronunciation accuracy 2) was entered in the final step, following the inclusion of predictors that had shown significant contributions. For each of the analyses, the pronunciation accuracy measure that contributed the most to the explained variance was used. Across all analyses, the percentage of acceptably pronounced words without prompting was a stronger predictor than the pronunciation accuracy measure that also included words correctly pronounced after prompting. Therefore, all analyses reported below included only the measure based on unprompted pronunciations (i.e., *pronunciation 1*).

In the L1 group, the measure of phoneme identification from the initial block of pretest measures was the strongest single predictor of both posttest word and non-word reading, explaining 11% and 17% of the variance, respectively (Table 3). After accounting for this contribution, none of the other predictors from the initial block contributed further. However, pronunciation accuracy added a significant 6% to the explained variance in posttest non-word reading (Model 2) but did not significantly improve the prediction of posttest word reading (Model 1).

**Table 3:** Prediction of reading of words and non-words at posttest in Grade 1 from predictors in kindergarten for L1 speakers (*n* = 68)

Dependent variable (Posttest Grade 1)	Model	Step	Predictor (Pretest kindergarten)	*R*²	∆*R*²	F change	β
Reading words	1	1 2	PA: Phoneme identification Pronunciation accuracy 1	0.11 0.14	0.11 0.03	7.78** 2.27	0.31* 0.18
Reading non-words	2	1 2	PA: Phoneme identification Pronunciation accuracy 1	0.17 0.23	0.17 0.06	13.52** 4.66*	0.39** 0.24*

*PA* phonological awareness. Standardized regression coefficients (*β*s) are provided for the final models

*p < .05. **p < .01

In the L2 group, letter knowledge emerged as the strongest predictor of posttest word reading, accounting for 36% of the variance (Table 4). Once this variable was considered, no other predictors from the initial block made a significant additional contribution. However, pronunciation accuracy significantly increased the explained variance by an additional 8% (Model 1). Regarding posttest non-word reading, letter-sound knowledge predicted 26% of the variance, with syllable identification contributing an additional 9% (Model 2). Adding pronunciation accuracy in the final step did not lead to a significant increase in the explained variance for non-word reading in this group.

**Table 4:** Prediction of posttest reading of words and non-words in Grade 1 from predictors in kindergarten for L2 learners (*n* = 33)

Dependent variable (Posttest Grade 1)	Model	Step	Predictor (Pretest kindergarten)	*R*²	∆*R*²	F change	β
Reading words	1	1 2	Letter knowledge Pronunciation accuracy 1	0.36 0.45	0.36 0.08	17.62** 4.51*	0.44** 0.33*
Reading non-words	2	1 2 3	LSK: Identifying letters PA: Syllable identification Pronunciation accuracy 1	0.26 0.35 0.38	0.26 0.09 0.03	11.05** 4.24* 1.29	0.35* 0.23 0.21

*PA* phonological awareness, *LSK* letter-sound knowledge. Standardized regression coefficients (*β*s) are provided for the final models. *p < .05. **p < .01

#### Prediction of responder status

Participants were classified as either poor or adequate responders based on their post-intervention outcome scores, following procedures established in prior research (see above). To establish an appropriate cut-off, we examined the score distributions for word and non-word reading. In both measures, 16 out of 101 participants read a maximum of three words correctly, suggesting minimal or no decoding ability. As a result, the 16 lowest-performing participants on the composite measure (derived from standardized posttest scores in word and non-word reading) were identified as poor responders, while the remaining 85 were classified as adequate responders. Among the L1 speakers, 15% were classified as poor responders, while 18% of the L2 learners fell into this category.

Logistic regression analyses were then conducted to address our research question: To what extent can pretest performance on the most predictive measures classify children with subsequent poor or adequate response to the intervention? Generally, logistic regression is used to predict a dichotomous outcome based on a set of independent variables. This analysis provides an estimate of the variance in the dependent variable explained by the predictors, typically reported as Nagelkerke *R²*. To further assess predictive accuracy, the area under the Receiver Operating Characteristic (ROC) curve is calculated for each logistic model. The ROC curve plots the true positive rate (sensitivity) against the false positive rate (1 – specificity) for all possible cut points. The area under the curve (AUC) serves as an overall indicator of classification accuracy (Swets, 1992). For practical purposes, AUC values of 0.90 or higher indicate excellent predictive accuracy, while values above 0.80 are considered good or acceptable (Hintze & Marcotte, 2010).

To assess how well the most predictive pretest measures classified children with poor and adequate response to the intervention, the pretest measures were initially entered forwards stepwise (based on Wald statistics). Letter-sound knowledge (identifying letters) emerged as the strongest individual predictor, accounting for 33% of the variance in responder status and yielding an AUC of 0.82 (Table 5, Model 1). After controlling for this variable, pronunciation accuracy was the only additional significant predictor, contributing an extra 10% to the explained variance and increasing the AUC to 0.86.

The final research question was: Does the inclusion of a measure of pronunciation accuracy add to the early classification beyond the other predictors? To further account for potential predictors, phonological awareness was included prior to adding pronunciation accuracy in the final step (Table 5, Model 2), given its strong predictive contribution to the overall variance (Table 3). The addition of phonological awareness after letter-sound knowledge increased the explained variance by 4%, but this improvement was not statistically significant (*p* = .08). Notably, even when pronunciation accuracy was entered after both letter-sound knowledge and phonological awareness, it still accounted for 9% of the variance in responder status.

To further explore the predictive value of pronunciation accuracy, a separate logistic regression analysis was performed (Table 5, Model 3), entering pronunciation accuracy first. In this model, pronunciation accuracy alone explained 31% of the variance and produced an AUC of 0.79. Letter-sound knowledge again emerged as the strongest predictor when the remaining measures were added stepwise, contributing an additional 12% to the explained variance.

**Table 5:** Prediction of responder status at posttest in Grade 1 from pretest measures in kindergarten

Model	Step	Predictor added at each step	Nagelkerke *R*²	Nagelkerke *R*² Change	χ² change	Wald (last step)	AUC
1	1 2	LSK: Identifying letters Pronunciation accuracy 1	0.33 0.43	0.33 0.10	21.41** 7.51**	7.42** 6.01*	0.82 0.86
2	1 2 3	LSK: Identifying letters PA: Phoneme identification Pronunciation accuracy 1	0.33 0.37 0.46	0.33 0.04 0.09	21.41** 3.05 6.87**	4.19* 2.25 5.59*	0.82 0.85 0.87
3	1 2	Pronunciation accuracy 1 LSK: Identifying letters	0.31 0.43	0.31 0.12	19.88** 9.03**	6.01* 7.42**	0.79 0.86

*PA* phonological awareness, *LSK* letter-sound knowledge, *AUC* area under the curve, from ROC-analyses

*p < .05; **p < .01

## Discussion

The purpose of this study was to examine how well pretest measures could predict at-risk children’s responses to an early reading intervention, allowing for a comparison of L1 speakers and L2 learners. In various combinations, the strongest predictors of overall variance in reading outcomes were found to be measures of phonological awareness, letter knowledge, letter-sound knowledge, and pronunciation accuracy. A relatively good classification of children with subsequent poor or adequate response to the intervention (as indicated by AUC values well above 0.80) could be obtained based on just a couple of pretest measures, including pronunciation accuracy.

Notably, this study provided evidence for the added predictive value of a measure of pronunciation accuracy to forecast reading outcomes of an early reading intervention and to identify children at increased risk of poor response, even after taking the children’s initial performance on measures of phonological awareness into account. This finding extends the result from the study by Elbro and Petersen (2004) who demonstrated that pronunciation accuracy contributed substantially to the prediction of at-risk children’s responsiveness to training in phoneme awareness. Our findings also extend the results from previous research documenting that pronunciation accuracy can add to the prediction of development of reading abilities beyond phonological awareness among at-risk children (Tambyraja et al., 2020). To our knowledge, this added value from pronunciation accuracy has not previously been documented in the specific context of predicting response to early reading intervention. This new finding is important because it may enhance our understanding of why some at-risk children seem resistant to evidence-based early reading interventions. This finding may also guide the development of support for children at-risk.

Although pronunciation accuracy explained a statistically significant unique proportion of variance, the magnitude of this added variance was relatively modest (approximately 6-10%). This suggests that pronunciation accuracy should not be viewed as replacing established predictors such as phonological awareness. However, in applied educational contexts, even small increments in prediction may be meaningful when identifying children at risk of poor response to intervention. Pronunciation accuracy may provide complementary information that refines risk estimates and, importantly, may help identify children whose difficulties are related to underspecified phonological representations. Such information could inform instructional planning by indicating a need for additional support targeting word-level phonological representations and pronunciation during intervention.

Broadly speaking, pronunciation accuracy depends on both articulatory control and on the quality of the speaker’s phonological representations of lexical items. Limited articulatory control in the context of well-specified phonological representations is a cause of the so-called “fish” phenomenon when a person can detect pronunciation errors, such as “fis” for *fish* or “Arfer” for *Arthur* but cannot correct them. To rule out problems with articulatory control, some studies of phonological representations have repeated the child’s pronunciation back to the child and asked the child whether the pronunciation is correct (e.g., Elbro et al., 1998; Anthony et al., 2010). In these and other studies, children rarely find anything wrong with their pronunciation; in the case they do, it is easy to detect a pattern (a “phonological process”) across multiple words that share particular sound segments. The findings from these studies point to poorly specified phonological representations of discrete lexical items as the primary source of difficulties with pronunciation accuracy, at least as a predictor of the development of phonological awareness and initial reading.

Remediation of poorly specified phonological representations is severely challenged by the fact that these representations are of *individual* lexical items. Hence, remediation of phonological representations faces challenges to the remediation of other vocabulary limitations, e.g., breadth of vocabulary knowledge. Intervention is unlikely to generalize easily from trained items to untrained ones (Elbro & Pallesen, 2002). One educational implication is that a focus on quality of phonological representations should be an integral part of early activities with words. Another implication is that children need to know fully specified pronunciations of words before the words are part of activities with phonological segments and their orthographic representations.

Interestingly, the overall prediction rate was higher for L2 learners than for L1 speakers, and the predictors differed somewhat among these groups. Obviously, words in an L2 are likely to be less well practiced and less well specified in the mental lexicon than words in an L1. This difference was masked by the difference in simple vocabulary knowledge in the current study. However, individual differences in pronunciation accuracy were clearly larger in the L2 group than in the L1 group pointing to the fact that the children in the L2 group differed greatly in pronunciation accuracy. A likely consequence is that *some* of the children in the L2 group were seriously challenged by underspecified phonological representations during the intervention.

Some of the pretest measures in our study turned out *not* to be significant predictors of reading outcomes. Importantly, this was the case for vocabulary. This result is in line with previous findings (Lam & McMaster, 2014) and suggests that the outcomes of this early reading intervention did not depend on a certain level of vocabulary in the language of instruction. Neither were pre-intervention word reading skills found to predict reading outcomes in this study although this has been found to be the case in some previous studies (Lam & McMaster, 2014). Given that children who could read generally were not included in this study, it was to be expected that word reading did not predict any variance in the present sample. It was more interesting that the dynamic test of decoding did not emerge as a significant predictor of reading outcomes although this test has previously been shown to be a good kindergarten predictor of later reading difficulties (Gellert & Elbro, 2017b, 2018). However, since the children in the current sample represented the lower end of the distribution, the test exhibited floor effects, limiting its effectiveness as a predictor for this sample. This finding indicates that measures that are useful for the identification of at-risk children and selection for intervention are not necessarily useful for the prediction of response to intervention.

Pronunciation accuracy did not correlate significantly with either vocabulary or phonological awareness in the L1 speakers, and only with phonological awareness in the L2 learners (Table 2). These results underscore the independence between the three constructs (with a possible exception). Pronunciation accuracy relates to the quality of phonological representations of lexical items, whereas vocabulary (usually) relates to the sheer number of representations in the mental lexicon. Phonological awareness affords access to and manipulation of the sound segments of phonological items. Obviously, this access depends on whether or not the particular segments are present in the phonological representations. However, by the age of approximately seven, children’s phonological representations are typically fully specified – at least when it comes to words that are typically used for reading instruction and training of phonological awareness.

The situation may be somewhat different in children who are L2 learners. They have varying experience with the L2 and may even experience interference from their L1 phonology. As a result, their pronunciation accuracy may be more individually variable than in their L1 peers, as seen in the results (Table 1). This means that some of the L2 learners’ lexical representations may still be phonologically underspecified and as such a poor foundation for phonological awareness. Other L2 learners may not experience a similar limitation. In consequence, a correlation between pronunciation accuracy and phonological awareness may show up in L2 learners – similar to what has been observed in younger L1 speakers (Elbro et al., 1998; Elbro & Pallesen, 2002). We prefer to remain cautious even though the pattern of correlations within each of the two groups of participants may look somewhat different (Table 2). We made no prior predictions of such differences, and given the small L2 group, the findings in this group and possible differences from the L1 group should be considered exploratory.

This study had some limitations, but also some possible implications for future practice.

First, while the whole sample was just above 100 children, the subgroup of L2 learners was relatively small (*n* = 33) for hierarchical linear regression analysis. Alternative analytic approaches might be considered, such as models with interaction terms. However, such models primarily address moderation rather than within-group prediction. In addition, given the relatively small size of the L2 sample, interaction effects would be expected to be estimated with limited statistical power and potentially unstable coefficients. Obviously, the findings particularly for the group of L2 learners need replication with a larger sample of L2 children. Additionally, because of the limited number of L2 learners, logistic regression analyses could not be conducted separately for this group.

Second, the experimental measure of pronunciation accuracy was not useful for assessing four children who could not name any of the pictures in the test. Unfortunately, this implicated that although this study included L2 learners, our analyses did not include the children with the most limited L2 vocabulary. The percentage of acceptably pronounced words without prompting turned out to be a stronger predictor than the pronunciation accuracy measure that also included pronunciation of words correctly named after prompting. Therefore, the analyses employed the measure based on only unprompted pronunciations. For some of the children, the employed measure of pronunciation accuracy was based on relatively few words which may have reduced the sensitivity of the measure. For future studies focusing on children with limited vocabulary in their L2 we recommend the inclusion of more common target words likely to be known by these children. Alternatively, the assessment of pronunciation accuracy could be based on, e.g., the percentage of consonants correctly articulated in a connected speech sample gathered during a story retell task, similarly to some previous studies (e.g., Tambyraja et al., 2020).

Third, in line with previous research (e.g., Hatcher et al., 2006; van Uittert et al., 2022), our study focused on children’s response to a specific intervention program. This program was based on international research and developed to represent a state-of-the-art intervention. Therefore, it is plausible that our findings can be generalized to other state-of-the-art interventions. However, replication in other countries is called for, particularly with respect to the added value of pronunciation accuracy for predicting response to early reading interventions.

Despite these limitations, the study may have important implications for future educational practice. The findings indicate that a small set of pre-intervention measures – including a pronunciation accuracy measure – can relatively reliably identify children at risk of struggling seriously with word decoding, even after receiving an evidence-based small group intervention like the one described in this study. Such information could help educators tailor instruction at an early stage. Depending on available resources, different implications could be considered for children predicted to be poor responders, such as prioritizing attention within the standard program, supplementing small group instruction with individual sessions, or referring them to a more specialized intervention aligned with their specific needs. However, decisions regarding determining intervention responsiveness should be made with caution. Further research is needed to establish evidence-based guidelines for predicting responsiveness and enhancing the implementation of future interventions.

## Data Availability

The data are not publicly available due to legal and ethical concerns.

## Appendix

**Table 6:** Reliability estimates for study measures and corresponding source references

Measure	Reliability estimate	Original validation study
Letter knowledge (K)	α ≥ 0.91 (Gellert et al., 2024)	Elbro & Petersen, 2004
Dynamic decoding 1: Learning novel letters (K)	α = 0.81 (Gellert & Elbro, 2018)	Elbro et al., 2012
Dynamic decoding 2: Learning to read (K)	α = 0.96 (Gellert & Elbro, 2018)	Elbro et al., 2012
Letter-sound knowledge (K)	α ≥ 0.84 (Present study)	Elbro & Petersen, 2004
Phonological awareness (K)	α = 0.86 (Present study)	Gellert & Elbro 2017a
RAN (K)	*r* = .63 (Juul et al., 2014)	Juul et al., 2014
Vocabulary (K)	α = 0.86 (Present study)	Gellert & Elbro, 2018
Word reading (K and G1)	Test-retest *r* = .82 (Gellert & Elbro, 2018)	Elbro & Petersen, 2004
Non-word reading (G1)	Correlation with word reading *r* = .86 (Present study)	Elbro & Petersen, 2004

*K* Kindergarten, *G1* Grade 1

**Table 7:** List of the 16 target words for assessment of pronunciation accuracy

Item	Danish target word (translation)
20	Helikopter (helicopter)
21	Flagermus (bat)
22	Blæksprutte (octopus)
23	Kænguru (kangaroo)
24	Skelet (sceleton)
25	Mikrofon (microphone)
26	Vulkan (volcano)
27	Skruetrækker (screwdriver)
28	Kaktus (cactus)
29	Violin (violin)
30	Labyrint (labyrinth)
31	Pyramide (pyramid)
32	Akvarium (aquarium)
33	Globus (globe)
34	Reol (bookcase)
35	Termometer (thermometer)

**Table 8:** Supplementary statistics for measures of pronunciation accuracy for participants with Danish as L1 and L2

Measure	Mean (standard deviation)	
	L1 (*n* = 68)	L2 (*n* = 33)
Pictures named correctly without prompting	10.47 (2.97)	6.21 (3.71)
Words pronounced acceptably without prompting	7.90 (2.86)	4.79 (3.07)
Pictures named correctly in total (with or without prompting)	12.54 (2.67)	8.15 (4.43)
Words pronounced acceptably in total (with or without prompting)	9.09 (2.85)	5.70 (3.46)
